# Synapse-specific Lrp4 mRNA enrichment requires Lrp4/MuSK signaling, muscle activity and Wnt non-canonical pathway

**DOI:** 10.1186/s13578-021-00619-z

**Published:** 2021-06-05

**Authors:** Hongyang Jing, Peng Chen, Tiankun Hui, Zheng Yu, Jin Zhou, Erkang Fei, Shunqi Wang, Dongyan Ren, Xinsheng Lai, Baoming Li

**Affiliations:** 1 School of Life Science, Nanchang University, Nanchang, 330031 China; 2 Institute of Life Science, Nanchang University, Nanchang, 330031 China; 3 Human Aging Research Institute, Nanchang University, Nanchang, 330031 China; 4 Department of Psychology and Institute of Brain Science, School of Education, Hangzhou Normal University, Hangzhou, 311121 China

**Keywords:** Lrp4, Neuromuscular junction, β-gal, Muscle activity, Wnt non-canonical signaling

## Abstract

**Background:**

The neuromuscular junction (NMJ) is a peripheral synapse critical to muscle contraction. Like acetylcholine receptors (AChRs), many essential proteins of NMJ are extremely concentrated at the postjunctional membrane. However, the mechanisms of synapse-specific concentration are not well understood; furthermore, it is unclear whether signaling molecules critical to NMJ formation and maintenance are also locally transcribed.

**Results:**

We studied the β-gal activity encoded by a lacZ cassette driven by the promoter of the *Lrp4* gene. As reported for *Lrp4* mRNA, β-gal was in the central region in embryonic muscles and at the NMJ after its formation. However, β-gal was no longer in the central areas of muscle fibers in Lrp4 or MuSK mutant mice, indicating a requirement of Lrp4/MuSK signaling. This phenotype could be rescued by transgenic expression of LRP4 with a transmembrane domain but not soluble ECD in Lrp4 mutant mice. β-gal and AChR clusters were distributed in a broader region in *lacZ/ECD* than that of heterozygous *lacZ/*+ mice, indicating an important role of the transmembrane domain in Lrp4 signaling. Synaptic β-gal activity became diffused after denervation or treatment with µ-conotoxin, despite its mRNA was increased, indicating synaptic Lrp4 mRNA enrichment requires muscle activity. β-gal was also diffused in aged mice but became re-concentrated after muscle stimulation. Finally, *Lrp4* mRNA was increased in C2C12 myotubes by Wnt ligands in a manner that could be inhibited by RKI-1447, an inhibitor of ROCK in Wnt non-canonical signaling. Injecting RKI-1447 into muscles of adult mice diminished Lrp4 synaptic expression.

**Conclusions:**

This study demonstrates that synapse-specific enrichment of Lrp4 mRNA requires a coordinated interaction between Lrp4/MuSK signaling, muscle activity, and Wnt non-canonical signaling. Thus, the study provides a new mechanism for Lrp4 mRNA enrichment. It also provides a potential target for the treatment of NMJ aging and other NMJ-related diseases.

**Supplementary Information:**

The online version contains supplementary material available at 10.1186/s13578-021-00619-z.

## Background

The neuromuscular junction (NMJ) is a chemical synapse between motor nerve terminals and skeletal muscle fibers. It is critical to motoneurons’ control of muscle contraction. Located in the middle of the muscle fibers, NMJs occupy only one-thousandth of a muscle fiber’s surface. Yet, essential proteins for NMJ structure and function are highly concentrated at the post-junctional membrane. For example, the acetylcholine receptor (AChR) is concentrated at 10–20,000/μm^2^, a hallmark of the NMJ [1, 2]. This is thought to be achieved by at least two mechanisms. First, AChR proteins or small AChR clusters distributed along the muscle membrane become aggregated in response to agrin, a factor released by motor nerves. Agrin binds to LRP4, activates MuSK, and via Dok7 and rapsyn, mediates AChR clustering [3–7]. Mutation of the gene encoding each of these proteins prevents mice from forming the NMJ [8–11]. On the other hand, synaptic proteins, including the AChR, are synthesized locally at the NMJ. For example, mRNAs of AChR subunits, rapsyn and AChE are enriched at the NMJ [12–18]. Studies using transgenic mice have shown that the regulatory elements of AChR genes can direct the expression of reporter genes at the NMJ [18–21]. These results strongly support the notion that AChR genes are transcribed locally in subsynaptic nuclei of multinucleated muscle fibers. However, mechanisms underlying the synapse-specific transcription remain unclear. Ectopic expression of agrin or MuSK increased AChR mRNAs in non-synaptic regions [22–24]; however, treatment of muscle cells with agrin increased AChRε mRNA but had little effect on AChRα and δ mRNA levels in vitro [25], suggesting the involvement of additional mechanisms.

Lrp4 is a member of the LDL receptor family. It possesses a large extracellular domain (ECD) consisting of one LDLα domain, four β-propeller domains, and a few EGF-like domains between LDLa and β-propeller domains; a transmembrane domain; and a small intracellular domain (ICD). In the absence of agrin, it could bind to MuSK to maintain a basal activity [5, 6]. The agrin–Lrp4 interaction increases its binding with MuSK and thus activates the kinase [4–6]. Being a receptor for agrin, it is required for nerve-induced clusters [8, 26, 27]. In addition, LRP4 null mice fail to form primitive, aneural AChR clusters (i.e., before innervation) [8], suggesting that it is necessary for early postsynaptic differentiation of NMJ. Intriguing, *Lrp4* mRNA is enriched in the central of muscle fibers in mouse E14.5 embryos and adults [8, 28]. An interesting question remains what the mechanisms that control the expression of Lrp4 are? Because aneural AChR clusters form in advance of innervation, LRP4 expression is likely to be regulated by a mechanism independent of neural agrin.

This study explored the mechanisms that control Lrp4 expression during development, after denervation, and in aged mice. Because of the lack of reliable antibodies for Lrp4, we studied *Lrp4-lacZ* mice where the *Lrp4* gene was replaced with a cassette containing the *lacZ* gene. Under the control of the *Lrp4*’s endogenous promoter, β-gal expression is thought to faithfully indicate where Lrp4 is expressed [29, 30]. We found that β-gal expression was concentrated in the middle regions where aneural AChR clusters are located at E13, i.e., before innervation [31, 32], suggesting an innervation-independent mechanism to restrict LRP4 expression within the central region. Such expression was diminished by Lrp4 or MuSK mutation, revealing a necessary role of Lrp4 and MuSK in Lrp4 expression in the central region of muscle fibers. After NMJ formation, β-gal expression was in a good registry with AChR clusters, indicating NMJ-specific activation of the Lrp4 promoter. Interestingly, the NMJ-specific β-gal expression in adult mice requires Lrp4, particularly the ECD and transmembrane domain of Lrp4 and neuronal activity. Finally, we showed that Lrp4 mRNA in C2C12 myotubes was increased by Wnt ligands, including Wnt3, Wnt3a, and Wnt5a. However, this effect was independent of the canonical pathway but was inhibited by RKI-1447, an inhibitor of non-canonical Wnt signaling [33, 34]. Injecting RKI-1447 into muscles of adult mice diminished Lrp4 synaptic expression. These results demonstrate that Wnt/ROCK non-canonical signaling increased the activity of Lrp4 promoter to promote mRNA expression. All together, synapse-specific Lrp4 mRNA enrichment requires Lrp4/MuSK signaling, muscle activity and may involve a Wnt non-canonical signaling pathway. We also posit that Lrp4-lacZ mice could serve as an informative mouse model to study synapse-specific mRNA enrichment.

## Result

### Synapse-specific distribution of Lrp4 β-gal in *Lrp4-lacZ* mice

**Fig. 1:**
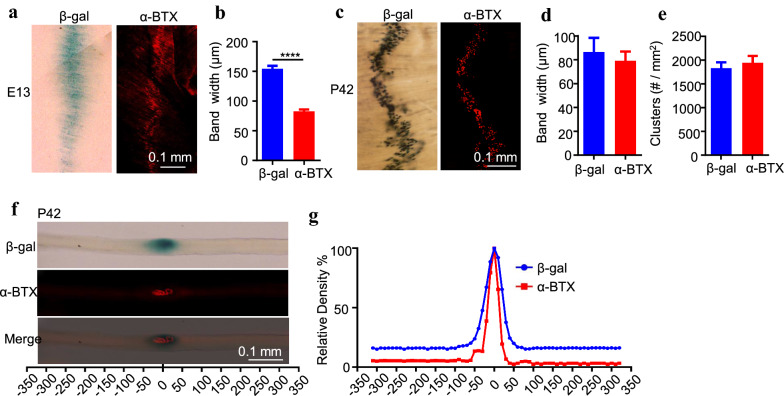
Synapse-specific distribution of Lrp4 β-gal in *Lrp4-lacZ* mice. **a**, **b** β-gal activity in the central region of muscle fibers. **a** Representative images of left ventral hemi-diaphragms of E13 lacZ/+ mice, which were stained whole-mount for β-gal activity (blue) and with Alexa fluor 594-conjugated α-BTX (referred to as α-BTX, red). Scale bar, 0.1 mm. **b** Central distribution of β-gal in lacZ/+ diaphragms. ****p < 0.0001, n = 7 mice per group, unpaired t-test. **c** Similar distribution of β-gal and AChR clusters in diaphragms at P42. Scale bar, 0.1 mm. **d**, **e** Quantification of the bandwidth of β-gal and AChR (**d**), the density of β-gal and AChR clusters (**e**). n = 6 mice per group, unpaired t-test. **f** Co-localization of β-gal cluster and AChR cluster in an EDL muscle fiber. Single muscle fibers of EDL were subjected to staining for X-gal and with α-BTX. Scale bar, 0.1 mm. **g** Superimposed β-gal and α-BTX intensity of single muscle fibers. Signals per 10 µm were averaged along muscle fibers by ImageJ and the central of AChR cluster was designated as point 0

Lrp4 protein is localized at the NMJ, and its mRNA is concentrated in the central region of muscle fibers [5, 8, 28]. To study underlying mechanisms, we took advantage of *Lrp4-lacZ* mice where the *Lrp4* gene was replaced with a cassette encoding a β-galactosidase (β-gal) [29, 30]. Under the control of the endogenous promoter, β-gal expression was expected to indicate where Lrp4 was expressed faithfully. Because two copies of the *Lrp4* gene were replaced by the cassette in homozygous *Lrp4-lacZ/Lrp4-lacZ* mice (referred to as *lacZ/lacZ*, unless otherwise specified), *lacZ/lacZ* mice failed to form AChR clusters and NMJs and died soon after birth (data not shown). Heterozygous *lacZ/*+ mice were able to form AChR clusters at E13 and were viable and fertile as wild-type mice. Interestingly, β-gal activity in *lacZ/*+ mice was distributed in the central region of muscle fibers (Fig. 1a), in agreement with Lrp4 protein and mRNA enrichment at the NMJ [5, 8, 28]. To determine the β-gal activity was truly at the NMJ, we developed a method to sequentially imaging AChR and β-gal activity in the same muscle samples to avoid the quenching effect of β-gal staining on the AChR signal (see “Methods”). At E13, both AChR clusters and β-gal activity were enriched in the middle region of diaphragms, although the area of β-gal appeared to be larger than that occupied by AChR clusters (Fig. 1a, b). This may be because of β-gal diffusion or poor innervation at this time and thus lack of muscle activity that may regulate synapse expression [31, 32]. Therefore, we examined β-gal in diaphragms of P42 mice. Interestingly, β-gal activity was distributed as aggregates, which colocalize with AChR clusters (Fig. 1c). The bandwidths and densities of β-gal clusters and AChR clusters were similar (Fig. 1d, e). To further confirm the co-localization of the β-gal cluster and AChR cluster, we assayed β-gal activity in individual muscle fibers and found it was present only at the NMJ (Fig. 1f, g). These results indicate that *Lrp4* mRNA was concentrated at the NMJ and suggested that Lrp4 β-gal is a reliable indicator of the promoter activity of the *Lrp4* gene [8, 28].

### Central localization of Lrp4 β-gal dependent on Lrp4/MuSK signaling

**Fig. 2:**
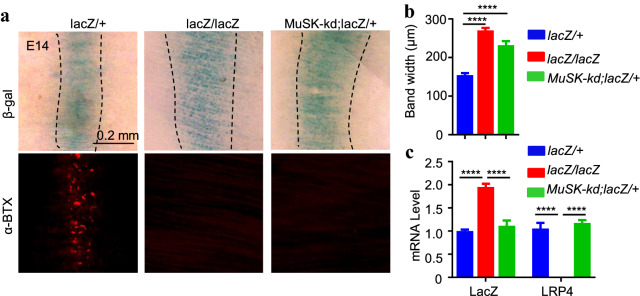
Requirement of Lrp4/MuSK signaling for central region localization of Lrp4 β-gal in mouse embryos. Diaphragms were isolated from E14 embryos of indicated genotypes. **a** Representative images. The increased bandwidth of Lrp4 β-gal in *lacZ/lacZ* and MuSK-kd;lacZ/+ mice. **b** Quantitative analysis of Lrp4 β-gal bandwidth. **c** The mRNA levels of *lacZ* and *Lrp4* in *lacZ/lacZ* and *MuSK-kd;lacZ/*+ mice. The transcription activity of the Lrp4 promoter didn’t require Lrp4 and MuSK in vivo. Data were shown as mean ± SEM, ****p < 0.0001, n ≥ 6 per group, one-way ANOVA

**Fig. 3:**
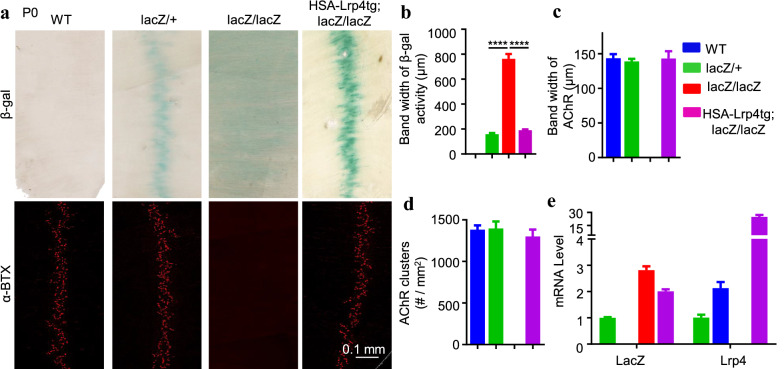
Requirement of LRP4 for Lrp4 β-gal at NMJ in neonatal mice. Diaphragms were isolated from P0 mice of indicated genotypes. **a** Representative images. Colocalizing β-gal with AChR in *lacZ/*+, but not *lacZ/lacZ* mice. Synaptic β-gal staining was restored in *lacZ/lacZ* mice by *HSA-Lrp4tg*. **b**–**e** Quantitative analysis of β-gal bandwidth (**b**), the bandwidth of AChR (**c**), AChR cluster density (**d**), and mRAN of *lacZ* and *Lrp4* (**e**). **a**–**e** ****p < 0.0001. n = 7–10 mice per group, one-way ANOVA. **e** n = 3 mice per group

To investigate mechanisms that regulate Lrp4 β-gal expression, we determined whether it required Lrp4/MuSK signaling pathway. As shown in Fig. 2, in *lacZ/*+ mice, the β-gal activity was localized in the central region of muscle fibers. But, the central bandwidth of β-gal activity was increased in lacZ/lacZ mice, indicating a requirement of Lrp4 signaling for β-gal central localization. Similar, widened β-gal distribution was observed in *MuSK-kd;lacZ/*+ mice carried a kinase-dead MuSK (Additional file 1: Figure S1). And this was not due to the change of *lacZ* mRNA levels (Fig. 2c). These results suggested Lrp4/MuSK signaling is required for the concentration of *Lrp4* mRNA at NMJ. To further test this hypothesis, we characterized diaphragms at P0 when AChR clusters are fully innervated in wild-type mice. At this time, β-gal activity was in the middle region of muscle fibers in *lacZ/*+ mice but completely diffused in *lacZ/lacZ* mice (Fig. 3a–d), again indicating the requirement of Lrp4. This phenotype was diminished after *lacZ*/+ mice were crossed with *HSA-Lrp4* mice that express wild type Lrp4 under the control of the HSA promoter [35]. This was not due to a change in lacZ mRNA levels (Fig. 3e). Together, these results indicate that synaptic distribution of *Lrp4* mRNA required Lrp4 and associated signaling in the NMJ development.

### A critical role of the transmembrane domain of Lrp4 for synaptic localization of Lrp4 β-gal

**Fig. 4:**
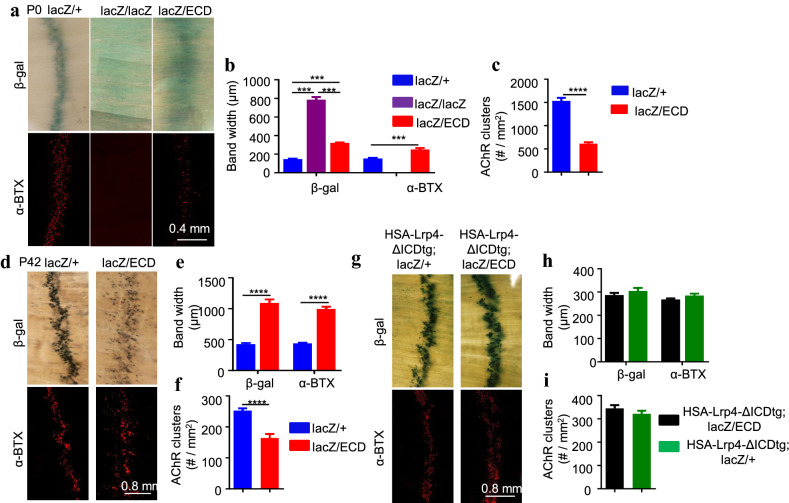
A critical role of the transmembrane domain of Lrp4 for synaptic localization of Lrp4 β-gal. **a**–**c** Inability of ECD to restore β-gal synaptic localization in P0 mice. **a** Representative images. **b** Quantitative analysis of bandwidth. **c** Quantitative analysis of AChR clusters. **d**–**f** Wider distribution of Lrp4 β-gal clusters in adult mice. **d** Representative images. **e** Quantitative analysis of bandwidth. **f** Quantitative analysis of AChR clusters. **g**–**i** Ability of Lrp4-ΔICD to restore Lrp4 β-gal synaptic localization in adult mice. **g** Representative images. **h** Quantitative analysis of bandwidth. **i** Quantitative analysis of AChR clusters. ***p < 0.001, ****p < 0.0001, n ≥ 7 mice per group, unpaired t-test

Lrp4 is a transmembrane protein with a large multi-domain ECD that interacts with agrin and MuSK. In vitro experiments showed that Lrp4 ECD was sufficient to enable agrin to activate MuSK in HEK293 cells, suggesting soluble ECD could function as a receptor [27, 36, 37]. To investigate how Lrp4 signaling regulates β-gal central localization, we crossed *lacZ/*+ mice with *ECD mutant* mice that express LRP4 ECD but without the transmembrane domain and ICD [26]. The resulting compound *lacZ/ECD* mice carried one copy *ECD* and one copy *lacZ*. Compared with *lacZ/lacZ* mice at P0, the bandwidth of the β-gal activity in *lacZ/ECD* mice was narrower (Fig. 4a, b). Concomitantly, AChR clusters were barely detectable in *lacZ/lacZ* mice but readily detectable in *lacZ/ECD* mice (Fig. 4a–c). However, compared with *lacZ/*+ mice that express one copy of wild type LRP4, the β-gal activity of lacZ/ECD distributed in a wider region, and the number of AChR clusters was much fewer (Fig. 4a–c). These results suggest that ECD partially rescued NMJ deficits and widened β-gal distribution by LRP4 mutation. At P42, the distribution of β-gal and AChR clusters was wider, and the cluster number smaller in *lacZ/ECD* mice than in *lacZ/*+ mice (Fig. 4d–f). These results suggest that full Lrp4 signaling cannot be mediated by ECD alone but requires the transmembrane domain. To test this hypothesis, we generated *HSA-Lrp4ΔICD* mice that express Lrp4 ECD and transmembrane domain (but without ICD). As shown in Fig. 4g, transgenic expression of Lrp4ΔICD was able to rescue the β-gal distribution phenotype in *lacZ/ECD* mice (Fig. 4g–i). These results suggest that the synaptic distribution of β-gal requires the signaling mediated by the ECD and transmembrane domain of Lrp4.

### Requirement of muscle activity for Lrp4 β-gal distribution at NMJs

**Fig. 5:**
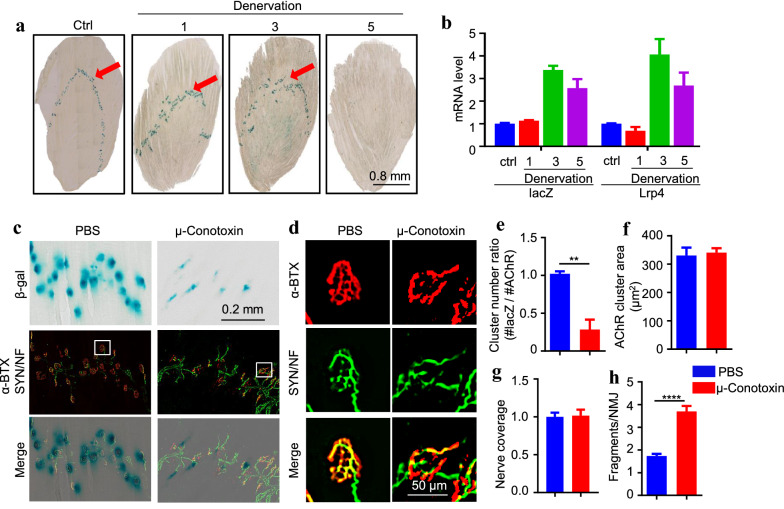
Requirement of muscle activity for Lrp4 β-gal concentration at NMJ. **a** Diffused synaptic Lrp4 β-gal in denervated TA muscles. Muscle sections (90 µm thick) were prepared from control or denervated lacZ/+ mice at P42 and were subjected to β-gal staining. Red arrow: NMJ localization. Scale bar, 0.8 mm. **b** Increased mRNA levels of Lrp4 and lacZ in denervated TA muscle. n = 3 mice per group. **c** Diffused synaptic Lrp4 β-gal in µ-conotoxin-injected muscles. TA muscles were injected with PBS or µ-conotoxin (80 µl, 10 µM) once every 2 days for 5 days. Slices (90 µm thick) were stained with α-BTX (red) and SYN/NF (green), and then subjected to X-gal staining (blue). Scale bar, 0.2 mm. **d** Enlarged images of AChR clusters from the box in **c**. Scale bar, 50 µm. **e** Decreased number of AChR clusters with Lrp4 β-gal cluster in µ-conotoxin-treated muscles. **p = 0.0058. **f** No change in AChR areas. p = 0.7489. **g** No change in nerve coverage (red/green). p = 0.8679. **h** Increased fragments of AChR clusters in µ-conotoxin-treated muscles. ***p < 0.0001. n = 4 mice per group, 30–40 NMJ per mice, unpaired t-test

Synaptic expression at the NMJ requires neuronal activity, which via releasing ACh controls muscle activity [38, 39]. For example, AChR expression is suppressed by muscle electric activity [40]. To determine whether Lrp4 mRNA synaptic distribution is regulated by neuronal activity, we studied the effect of denervation. As shown in Fig. 5a, β-gal activity was confined to the middle of muscle fibers of TA muscles in control mice but gradually diffused after denervation at day 3 after denervation. The β-gal activity was not detectable by staining in muscles at day 5 and afterward. This reduction was not due to the reduced level of *lacZ* mRNA, which was in fact increased in denervated muscles of *lacZ/*+ mice beginning at day 3 (Fig. 5b). In particular, *lacZ* mRNA peaked at day 3 when β-gal activity at the NMJ began to reduce. In addition, denervation didn’t affect synaptic nuclei aggregation (Additional file 1: Figure S2). These results suggest that muscle activity could suppress the *Lrp4* promoter activity but is required for synaptic *Lrp4* mRNA. To further test this hypothesis, we studied the effect of μ-conotoxin, a toxin that inhibits muscle activity by blocking muscle-specific voltage-gated sodium channels [41]. The TA muscles of adult *lacZ/*+ mice were injected with μ-conotoxin (80 μl, 5 μM for twice, at 1st day and 3rd day), where control muscles were injected with PBS. In μ-conotoxin treated TA muscle, the CMAP amplitude of 1st stimulation decreased, and muscle tetanic force decreased by electrical-stimulated muscle, but the CAMP amplitude ratio of 1st/10th was not changed compared with the control group (Additional file 1: Figure S3). These indicate μ-conotoxin blocks muscle excitability but does not affect synapse transmission. However, β-gal activity at the NMJ was dramatically reduced in TA muscles of toxin injected *lacZ/*+ mice (Fig. 5c). The number of β-gal clusters, but not AChR clusters, was reduced, leaving many AChR clusters without β-gal staining (Fig. 5c, e). Notice that μ-conotoxin did not affect the size of AChR clusters (Fig. 5f) and their innervation (Fig. 5g). Nevertheless, in toxin injected *lacZ/*+ mice, AChR clusters appeared to be more fragmented (Fig. 5h). Together, these results are in agreement with the notion that β-gal synaptic distribution requires muscle activity.

**Fig. 6:**
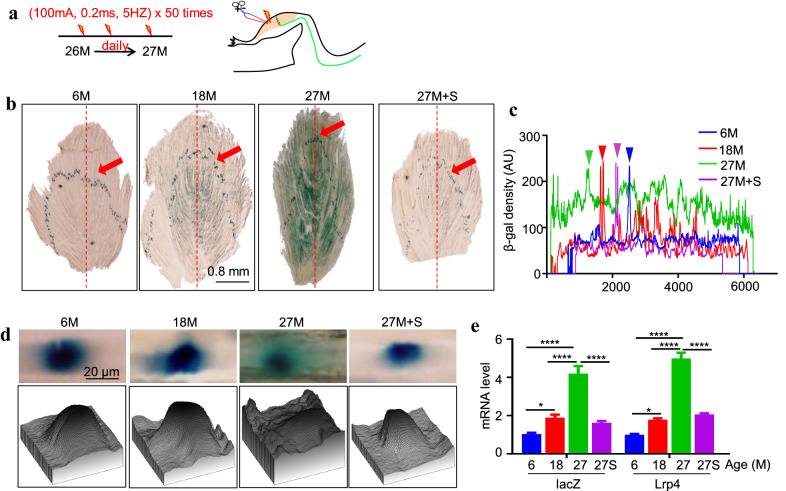
A necessary role of muscle activity for Lrp4 β-gal synaptic localization in aged mice. **a** Scheme of electric stimulation of muscle. **b** Lrp4 β-gal was diffused in aged muscles but became localized at NMJ after electric stimulation of muscle. Red arrow: NMJ localization. Scale bar, 0.8 mm. **c** Quantification of Lrp4 β-gal across muscle fibers (the red dotted line of **b**). Triangle indicates NMJ localization. **d** Synaptic Lrp4 β-gal distribution revealed by surface plot analysis. Scale bar, 20 µm. **e** Increased mRNA of lacZ and Lrp4 in aged muscles but reduced by stimulation. *p < 0.05 (*lacZ = 0.019, *Lrp4 = 0.0398), ****p < 0.0001. n = 4 mice per group, one-way ANOVA. M: month; S: stimulation; 27S: 27M+S

We also studied the effect of aging on Lrp4 β-gal expression in aged mice because aging is associated with a NMJ decline [3]. As shown in Fig. 6, the β-gal activity was detected only at NMJ at 6 months (M) of age. At 18 M, the β-gal activity became detectable in regions outside of the NMJ. At 27 M, the β-gal activity was diffused and detected in the non-synaptic region (Fig. 6b–d). This is associated with an increase in mRNA (Fig. 6e). To determine this was due to reduced muscle activity, we stimulated TA muscles of 26 M-old mice with electricity (100 mA, 0.2 ms, 1 s, 5 Hz × 50 times per day) for 30 days. Remarkably, the β-gal activity in the non-synaptic region was decreased but increased in NMJ (Fig. 6d). These results indicate Lrp4 β-gal synaptic enrichment requires muscle activity.

### Wnt promotion of *Lrp4* expression by a non-canonical pathway

**Fig. 7:**
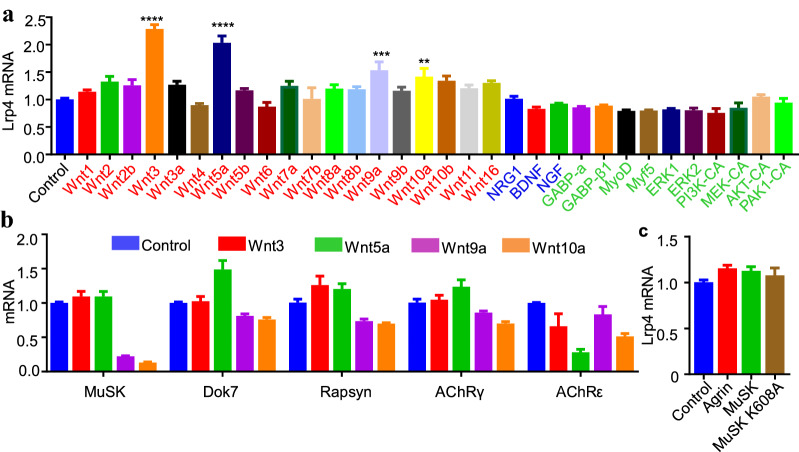
Increased Lrp4 mRNA expression by Wnt ligands. C2C12 myoblasts were transfected with plasmids. The resulting myotubes were subjected to qRT-PCR. **a** *Lrp4* mRNA increased by Wnt3, wnt3a, wnt5a, wnt9a, and wnt10a. **b** Effects of Wnt ligands on MuSK, Dok7, rapsyn, and AChRγ- and ε-subunits. **c** No effect of agrin or MuSK or its mutant on LRP4 expression. **p = 0.0038 ***p = 0.001, ****p < 0.0001, n ≥ 4, one-way ANOVA

**Fig. 8:**
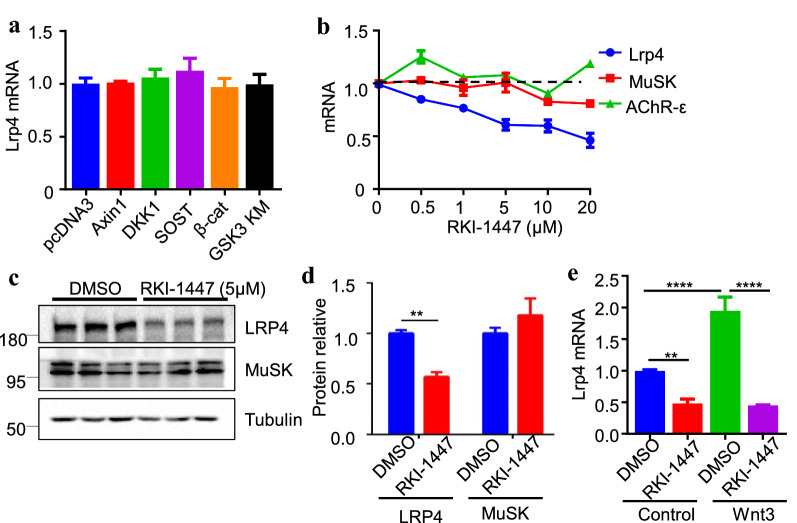
Inhibition of Lrp4 expression by ROCK inhibitor RKI-1447. **a** No effect of canonical Wnt signaling molecules on Lrp4 mRNA levels. n > 4, one-way ANOVA. **b** Reduced Lrp4 mRNA by RKI-1447. Myotubes were treated with RKI-1447 for 8 h. n > 3. **c**, **d** Reduced Lrp4 protein in RKI-1447-treated C2C12 myotubes. **c** Representative blots. **d** Quantification of data in **c**. **p = 0.0016, n = 3, unpaired t-test. **e** Inhibition of Lrp4 expression in Wnt3-expressing C2C12 myotubes. **p = 0.0047, ****p < 0.0001, n ≥ 6, one-way ANOVA

To understand regulatory mechanisms of *Lrp4* expression, we screened for factors that could enhance mRNA levels of *Lrp4* in muscle cells. We focused on factors that have been implicated in NMJ formation. Noticeably, *Lrp4* mRNA levels were increased by several Wnt ligands, including Wnt3, Wnt5a, Wnt9a, and Wnt10a (Fig. 7a). This effect appeared to be specific because *Lrp4* mRNA levels were not enhanced by neuregulin 1 (Nrg1), Bdnf, Ngf, Gabp-a, Gabp-β1, MyoD, Myf5, ERK, PI3K, MEK, AKT, and PAK1 pathway (Fig. 7a). In addition, Wnt3 did not affect mRNA levels of MuSK, Dok7, rapsyn, AChRγ, and AChRε (Fig. 7b). Moreover, *Lrp4* mRNA was also not increased by neuron agrin stimulation or the expression of MuSK or the kinase-dead mutant K608A (Fig. 7c). These results suggest that Wnt ligands could increase lrp4 expression. To determine which intracellular pathways are required, we first overexpressed proteins known to regulate the β-catenin signaling, including Axin1, GSK-3B-KM (a kinase-deficient mutant), or β-catenin in C2C12 myotubes. We found *Lrp4* mRNA was not altered by their expression (Fig. 8a), suggesting *Lrp4* expression may not be regulated by the canonical pathway. Next, we treated C2C12 myotubes with RKI-1447, an inhibitor of ROCK1/2, implicated in the Wnt non-canonical pathway [42–44]. It dose-dependently reduced *Lrp4* mRNA levels (Fig. 8b). As a control, RKI-1447 did not affect mRNA or protein levels of MuSK (Fig. 8c, d). Finally, RKI-1447 was able to inhibit the Wnt3-stimulated expression of Lrp4 (Fig. 8e). These results demonstrate that Lrp4 expression is regulated by Wnt signaling, possibly via the non-canonical pathway.

### Disruption of Lrp4 β-gal synaptic expression by RKI-1447

**Fig. 9:**
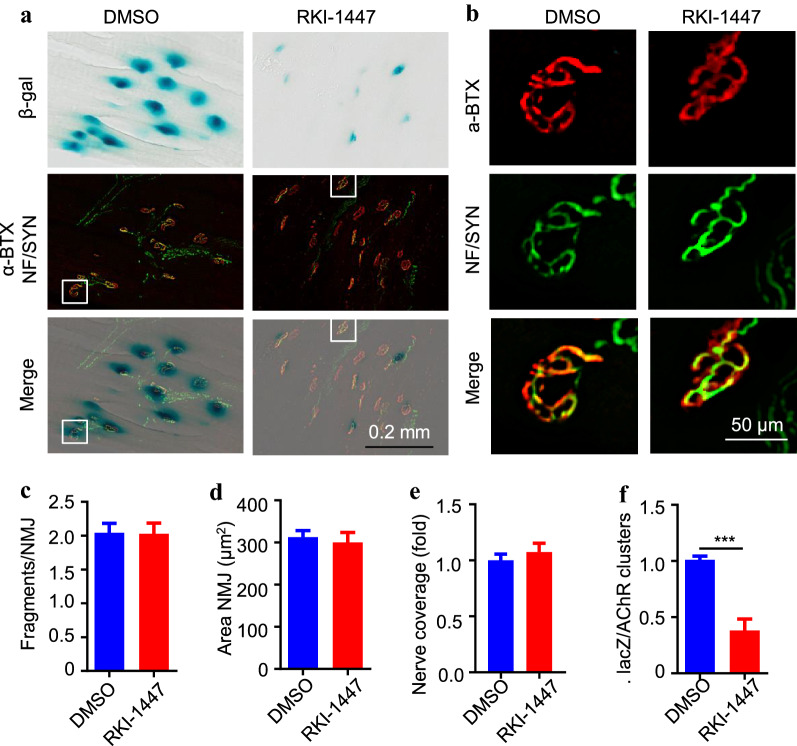
Disruption of Lrp4 β-gal synaptic expression by RKI-1447. TA muscles of adult lacZ/+ mice were injected with RKI-1447 or DMSO as control. **a** Representative images of β-gal, α-BTX (red) and SYN/NF (green). Scale bar, 0.2 mm. **b** Enlarged images of the box in (**a**). Scale bar, 50 µm. **c**–**e** No change in AChR cluster fragments (**c**), AChR area (**d**) and never coverage (**e**). **f** Decreased AChR clusters with β-gal activity. ***p = 0.0003. n > 4 mice per group, 30–40 NMJs per mice, unpaired t-test

To determine whether the non-canonical pathway contributes to Lrp4 synaptic expression in vivo, RKI-1447 (80 µl, 10 µM for twice, 1st day and 3rd day) into TA muscles of adult *lacZ/*+ mice for 5 days. Contralateral TA muscles were injected same volumes of vehicle (5% DMSO) as control. Five days after injection, TA muscles were first co-stained with Alexa Fluor™ 594-conjugated α-BTX and antibodies against SYN/NF and then stained for β-gal activity (see “Methods”). As shown in Fig. 9, RKI-1447 did not affect the fragments (Fig. 9a–c), size of AChR clusters (Fig. 9b, d) or their innervation (Fig. 9b, e). However, β-gal activity was dramatically decreased in RKI-1447-injected TA muscles compared with control. Many AChR clusters were left without β-gal activity (Fig. 9a, f), indicating that Lrp4 synaptic expression requires the non-canonical pathway of Wnt signaling.

## Discussion

By studying the β-gal expression of Lrp4 lacZ mice, we demonstrate that synaptic specific Lrp4 mRNA enrichment requires the Lrp4/MuSK signaling, muscle activity, and Wnt non-canonical signaling. In brief, Lrp4/MuSK signaling and muscle activity are responsible for the localization of Lrp4 mRNA, and Wnt non-canonical signaling promotes Lrp4 mRNA expression.

This study, we use β-gal activity to indicate the Lrp4 mRNA in Lrp4-lacZ mice where the Lrp4 gene was replaced with the lacZ gene. First, under the control of the endogenous promoter, β-gal expression indicated Lrp4 mRNA expression. This has been reported in a previous paper [30]. Secondary, in E14 and P0 lacZ/+ mice, the distribution of β-gal activity in the diaphragm was consistent with the previously reported result of Lrp4 mRNA in situ hybridizations (Figs. 2a, 3a) [8]. Finally, in diaphragms of P0 lacZ/lacZ mice or denervated TA muscle of adult lacZ/+ mice (Figs. 3a, 5a), the mRNA level of lacZ didn’t decrease, but the β-gal cluster was diffused. And in AChRα-nlacZ transgenic mice, min AChRα promoter-driven nls-lacZ was detected in all muscle nuclei [45]. These results suggest the localization β-gal in lacZ/+ mice is determined by the promoter or 5ʹ UTR of the Lrp4 gene, not due to the characteristics of the β-gal protein. And these also indicate β-gal was able to faithfully indicate the expression and localization of Lrp4 mRNA in Lrp4-lacZ mice.

Lrp4 is a transmembrane protein with a huge ECD containing an LDLa domain and four β-propeller domains. Agrin binds to the β1 domain to form a binary complex, two of which form a tetramer (i.e., two agrin and two Lrp4) to activate MuSK [4–6]. The tetrameric complex is essential for MuSK activation [4], likely by promoting the interaction with MuSK (via LRP4’s β3 domain) [5, 6]. Interestingly, soluble ECD of Lrp4 is sufficient to serve as agrin’s receptor to stimulate MuSK [27, 36]. Likewise, genetic rescue experiments show that NMJ deficits of Lrp4 null mice could be diminished by transgenic expression of a mutant Lrp4 without the intracellular domain (ICD) [26, 35]. These results suggest that the ICD of Lrp4 is unnecessary for agrin signaling, although it contains a characteristic NPXY motif [3, 46]. In agreement, mutant mice that express the Lrp4 ECD (i.e., LRP4 without the transmembrane domain and ICD) could survive to five months, unlike null mice that die soon after birth [26]. NMJ formation is retarded in ECD/ECD mice, with AChR clusters distributed across a broader region of muscle fibers, prolonged nerve branches, and smaller AChR clusters [26]. We show that the β-gal activity in *lacZ/*+ mice was localized in the central region in mouse embryos and at the NMJ in adult mice (Fig. 1). However, the β-gal activity was diffused in *lacZ/lacZ* mice before and after innervation (although with increased β-gal mRNA level), indicating that Lrp4 is required for the central localization of Lrp4 (Figs. 2 and 5). This phenotype was rescued in *Lrp4ΔICDtg* mice, suggesting that the ICD is dispensable (Fig. 4). Interestingly, synaptic β-gal expression was not fully rescued by ECD expression; the bandwidth of β-gal and AChR remained larger in *lacZ/ECD* than *lacZ/*+ mice. These results indicate that the transmembrane domain of Lrp4 is critical to synapse-specific transcription.

Synapse-specific transcription is regulated by muscle activity. Reducing muscle activation by denervation, for example, increases the level of NMJ-specific mRNAs in non-synapse regions [13, 14, 40]. A current model of synapse-specific transcription is that muscle activation, in response to nerve stimulation, suppresses the gene expression in entire muscle fibers. However, gene expression is maintained at the NMJ because of local activation of the agrin-Lrp4–MuSK signaling [3, 38, 47]. We found that Lrp4 β-gal was increased at the NMJ 1 and 3 days after denervation (Fig. 5a) but became undetectable at the NMJ 5 days after denervation, although both *Lrp4* mRNA and *lacZ* mRNA were increased (Fig. 5b). This result suggests that muscle activity may play an active role in synapse-specific transcription. This notion is supported by data from experiments with µ-conotoxin, which reduced β-gal activity at the NMJ (Fig. 5c) but didn’t affect the transmission of NMJ (Additional file 1: Figure S3). This also suggests agrin–Lrp4–MuSK signaling doesn’t regulate Lrp4 gene expression, which is consistent with the results of changing the MuSK activity in C2C12 (Fig. 7c). In addition, in aged (27 mo) mice, LRP4 β-gal was diffused into entire muscle fibers, and NMJ localization became murky (Fig. 6). Remarkably, in vivo muscle stimulation restored β-gal NMJ localization. These results demonstrate that muscle activity may play an active role in regulating NMJ-specific Lrp4 mRNA enrichment. And they also suggest that reduced synapse-specific Lrp4 mRNA enrichment in aging may benefit by increasing muscle activity or exercise.

Wnt signaling has been implicated in NMJ formation [3, 38, 48, 49]. Wnt ligands can induce AChR clusters and potentiate agrin-induced formation of AChR clusters in cultured muscle cells [48, 49]. These effects seem to require the cysteine-rich domain (CRD) of MuSK, whose deletion impairs in vivo NMJ formation [4, 50–53], although this notion was challenged by another study [54]. MuSK interacts with disheveled (Dvl1), the critical scaffold protein in the Wnt signal pathway [55]. This interaction is required for forming the NMJ in vitro [55] and for axon guidance to the middle region of muscle fibers [53]. Mutant mice lacking or overexpressing β-catenin, a key mediator of the Wnt canonical pathway, in muscle fibers display both pre-and postsynaptic deficits [56–58]. Motoneuron-specific mutation of Wls, a protein necessary for Wnt cell surface [59], reduces synaptic junctional folds, increases AChR cluster fragments, and impairs presynaptic nerve terminals [60]. This study provides evidence that Wnt signaling contributes to synapse-specific localization of Lrp4 β-gal. First, Wnt ligands including Wnt3, Wnt5a, Wnt9a, and Wnt10a increase Lrp4 mRNA in cultured muscle cells (Fig. 7a). Second, Lrp4 mRNA was not altered by expressing regulators of Wnt canonical signaling such as β-catenin (Fig. 8a). Third, however, Lrp4 mRNA, either basal or Wnt3-induced, was reduced in muscle cells treated with RKI-1447, an inhibitor of the non-canonical ROCK pathway (Fig. 8e). Finally, Injecting RKI-1447 into muscles of adult mice diminished LRP4 synaptic expression (Fig. 9a). These results indicate Wnts promote Lrp4 expression via the non-canonical ROCK pathway.

In NMJ, some protein concentrations are due to the mRNA enrichment, like Lrp4, MuSK, AChRα, AChRδ, AChRε, etc. But the mechanism is not all the same [61]. For mRNA expression, AChRε is only expressed in synaptic nuclei, but Lrp4, MuSK, AChRα are expressed in synaptic nuclei and non-synaptic nuclei [45, 62]. Agrin increases AChRε expression but does not affect other NMJ critical genes [22]. GABPα/β regulated AChRδ and Utrophin mRNA expression [63, 64]. But our data show it didn’t affect Lrp4 mRNA expression. In our study, Wnt3 promotes Lrp4 expression. This effect was specific because Wnt3 did not affect mRNA levels of MuSK, Dok7, rapsyn, AChRγ and ε (Fig. 7b). And blocking ROCK with RKI-1447 decreased Lrp4 expression in C2C12 myotubes, but does not affect MuSK and AChRε (Fig. 8b). For mRNA localization, the mRNA distribution of MuSK, AChRα and δ was diffused in LRP4 mutant mice [8]. This phenotype is similar to the β-gal distribution in lacZ/lacZ mice (Figs. 2a, 3a). This indicated synapse-specific mRNA enrichment of MuSK, AChRα and δ required Lrp4 signaling. Here, we demonstrated the evidence of synapse-specific Lrp4 mRNA enrichment. LRP4/MuSK signaling regulated NMJ formation and maintenance to control muscle activity through NMJ. And then, muscle activity regulated Lrp4 mRNA enrichment in NMJ. This mechanism may also apply to the localization of NMJ other gene mRNAs.

## Conclusions

We report that synaptic-specific enrichment of Lrp4 requires the Lrp4/MuSK signaling, muscle activity, and Wnt non-canonical signaling. The role of muscle activity in regulating NMJ-specific mRNA enrichment suggests that reduced synapse-specific mRNA enrichment in aging may benefit by increasing muscle activity or exercise. Thus, it provides a potential treatment of NMJ aging and other NMJ-related diseases.

## Methods

### Mouse strains

*LRP4 ECD*, *HSA::LRP4-FL*^*Tg*^, and *HSA::LRP4-ΔICD*^*Tg*^ were described previously [26, 35]. The following mice were described previously *LRP4-LacZ* with genotyping primers being: 5ʹ-TTC TGC CCA GGA ATA GCC AG-3ʹ and 5ʹ-TGA GCG AGT AAC AAC CCG TC-3ʹ (KOMP, stock #VG15248) [30], *LRP4-ECD* with genotyping primers being: 5ʹ-CTC CAA TTT CCT GTC CCT TG-3ʹ, 5ʹ-GCC AGA GGC CAC TTG TGT AG-3ʹ and 5ʹ-CTG CAG CAG AGC TGA GGT TA-3ʹ [26]*, HSA::LRP4-ΔICD*^*Tg*^ and *HSA::LRP4-FL*^*Tg*^ [35]. *MuSK-K608A* (*MuSK-kd*) mice were generated by CRISPR–Cas9 and contain a K608A mutation—Lys608 (K608) to Analine (A), with genotyping primers being: 5ʹ-CAG GCT AAC CAG TAG GAG GTT ACA-3 and 5ʹ-GAG AGG AAG AGA CAT ATC GCA CTG-3ʹ and sequencing the PCR products to identify the genotype. *HSA::LRP4-FL*^*Tg*^ mice (LRP4-FLtg) expressed Flag-LRP4 in muscles under the control of the promoter of human skeletal a-actin (HSA) with genotyping primers being: 5ʹ-AAG AAA GAG GGT GGA CCT GAC-3 and 5ʹ-ACT GCT TCC TTC ACG ACA TTC-3ʹ. *HSA::LRP4-ΔICD*^*Tg*^ mice (LRP4-*ΔICDtg*) expressed Flag-LRP4 without the intracellular domain under the control of the HSA promoter, with genotyping primers being: 5ʹ-TGC CCA CCA CCT TAC ATT CT-3ʹ and 5ʹ-GAA CTG CTT CCT TCA CGA CAT-3ʹ. Mice were housed in a room with a 12-h light/dark cycle with ad libitum access to water and rodent chow diet.

### Chemicals

Chemicals and reagents were purchased from the following companies: 5-bromo-4-chloro-3-indoly β-d-galactopyranoside (β-gal, B4252) from Sigma-Aldrich (Poole, UK); RKI-1447 (S7195) from Selleckchem (Houston, TX, USA) and Alexa Fluor™ 594-conjugated α-BTX (#B13423, 1:3000 for staining) from Thermo Fisher (Lafayette, Colorado, USA). Information of antibodies was as follows: synapsin (D12G5, 1:1000 for staining); neurofilament (Millipore, AB1991; 1:1000 for staining); LRP4 (ECD) clone N207/27 (UC Davis/NIH NeuroMab Facility; #75-221, 1:500 for western blotting) [27, 29, 30, 65]; LRP4(ICD) clone N164/6 (UC Davis/NIH NeuroMab Facility; #73-182, 1:500 for western blotting); MuSK (1:1000 for western blotting) [5]; α-tubulin (Santa Cruz Biotechnology, sc-23948, 1:2000 for western blotting); Alexa Fluor 488-conjugated goat anti-rabbit IgG (Invitrogen, A-11034, 1:1000 for staining); horseradish peroxidase (HRP)-conjugated goat anti-rabbit IgG (Invitrogen, 31460, 1:2000 for western blotting) and goat anti-mouse IgG (Invitrogen, 31430, 1:2000 for western blotting) secondary antibodies.

Constructs expressing Flag-tagged Agrin, MuSK, LRP4, Wnt2, Wnt2b, Wnt3, Wnt3a, Wnt5a, Wnt5b, Wnt7a, Wnt8a, Wnt8b, Wnt9a, Wnt9b, Wnt10, Wnt10b, Wnt11 and Wnt16 were described previously [60, 66]. Constructs expressing HA-tagged Wnt1, Wnt4, Wnt6 and Wnt7b were generously provided by Dr. Xi He [60].

### Immunohistochemistry (IHC)

Diaphragms were dissected out and fixed with 4% paraformaldehyde (PFA) for 20 min at 4 ℃. After washing with phosphate-buffered saline (PBS) 3 times each for 30 min at room temperature (RT), diaphragms were treated with 0.1 M glycine for 1 h at RT and washed 3 times with PBS containing 0.5% Triton X-100, each for 30 min at RT. After incubating with the blocking buffer containing 5% goat serum, 2% BSA, 0.2% Triton X-100, 0.1% NaN3 in PBS, pH 7.4 for 3–4 h at RT or overnight at 4 ℃, samples were incubated with primary antibodies in the blocking buffer for 24 h at 4 ℃. Then samples were washed with PBS containing 0.5% Triton X-100 3 times, each for 30 min at RT, and mounted on gelatin-coated slides with Hydromount (National Diagnostics, HS-106). Z serial images were collected with an Olympus FSX100 fluorescence microscope (Olympus, Tokyo, Japan).

### X-gal staining

The anterior tibialis muscle was fixed in 4% PFA in PBS (with 2 mM MgCl_2_) for 20 min (TA muscles) at 4 ℃. After dehydration by 30% sucrose, muscles were frozen, cut into 80 μm sections, and placed on gelatin-coated glass slides (Sangon Biotech, Shanghai, China). Diaphragms were fixed for 15 min and placed on gelatin-coated glass slides. Muscle sections and diaphragm (whole mount) were then washed with PBS/2 mM MgCl_2_ for 10 min at 4 ℃, and incubated with the permeabilizing buffer containing 2 mM MgCl_2_, 0.01% sodium deoxycholate, 0.02% NP-40 in 0.1 M phosphate buffer, pH 7.4 for 10 min at 4 ℃. Samples were incubated with the staining solution (5 mM potassium ferrocyanide, 5 mM potassium ferricyanide, 2 mg/ml X-gal in the permeabilizing buffer) at 37 ℃ overnight. It is important to keep pH at 7.4, which is optimal for recombinant b-gal, but not for endogenous b-gal in mammalian tissues whose optimal pH is acidic [67]. Samples were washed with PBS for 4–8 h at 37 ℃ or performed IHC, mounted, and subjected to Z-stack imaging. In some experiments, single muscle fibers were isolated by incubating muscles in 0.2% collagenase type I at 37 ℃ for 1 h with occasional shaking [68]. Dissociated muscle fibers were collected and fixed with 2% PFA for 5 min at RT, placed on gelatin-coated slides, and stained for X-gal and AChR.

### Cell culture

Mouse C2C12 muscle cells and HEK293 cells were maintained as described previously [69]. They were transfected with 1 mg/ml PEI (polyethyleneimine, MW 40000, Polysciences, #24765) in DMEM, as described before [27, 69].

### Real-time PCR

Total RNA of muscles or C2C12 cells was extracted using Trizol (Sigma, #15596018) and transcribed into cDNA templates using High-Capacity cDNA Reverse Transcription Kits (Sigma, #4368813) following manufacturer’s instructions. Quantitative PCRs were run by StepOnePlus™ Real-Time System (Applied Biosystems, Foster City, CA, USA) using PowerUp™ SYBR Green Master Mix (Applied Biosystems, Life Technologies, Austin, TA, USA). The primers for specific genes were as follows: LRP4 (F: 5ʹ-GTG TGG CAG AAC CTT GAC AGT C-3ʹ, R: 5ʹ-TAC GGT CTG AGC CAT CCA TTC C-3ʹ); MuSK (F: 5ʹ-CTG AAG GCT GTG AGT CCA CTG T-3ʹ, R: 5ʹ-TCC TTT ACC GCC AGG CAG TAC T-3ʹ); Rapsyn (F: 5ʹ-GTG GAT GAA GGT GCT GGA GAA G-3ʹ, R: 5ʹ-CCG AGC AGT ATC AAT CTG GAC C-3ʹ); AChRε (F: 5ʹ-AGA CCT GAG GAC ACT GTC ACC A-3ʹ, R: 5ʹ-TCG TCC TTG CTG TAG TTG AGC C-3ʹ); AChRγ (F: 5ʹ-CTT GTG GCT AAG AAG GTG CCT G-3ʹ, R: 5ʹ-GCA AGG ACA CAT TGA GCA CGA C-3ʹ); LacZ (F: 5ʹ-AAT CTG TCG ATC CTT CCC GC-3ʹ, R: 5ʹ-TTA GCG AAA CCG CCA AGA CT-3ʹ); GAPDH (F: 5ʹ-GTG AAG GTC GGT GTG AAC GG-3ʹ, R: 5ʹ-CAA GCT TCC CAT TCT CGG CCT-3ʹ).

### Western blot analysis

Lysates were prepared in RIPA buffer (50 mM Tris–HCl, pH7.4, 150 mM NaCl, 2 mM EDTA, 1% sodium deoxycholate, 1% SDS, 1 mM PMSF, 50 mM sodium fluoride, 1 mM sodium vanadate, 1 mM DTT and protease inhibitor cocktails), as previously described [70, 71]. Samples were resolved on SDS-PAGE and transferred onto nitrocellulose membranes, which were incubated with Tris-buffered saline (TBS) with 0.1% Tween-20 (TBST) and 5% skim milk for 1 h at RT. Membranes were washed three times for 10 min before incubation with a primary antibody overnight at 4 ℃. After washing with TBST for three times, membranes were incubated with TBS containing HRP-conjugated secondary antibody for 1 h at RT. Immunoreactive bands were captured by Gel Doc-XR (Bio-Rad, Hercules, CA, USA) and band density was quantified by ImageJ.

### Denervation

Denervation was performed as described previously [72]. Briefly, adult mice were anesthetized with ketamine and xylazine cocktail (100 and 10 mg/kg body weight, respectively, i.p.). Disinfect and remove the hair between the thigh and the spinal cord and cut the skin to expose the sciatic nerve. The right sciatic nerve was removed a segment (5 mm), and the left sciatic nerve was subjected sham operation. Suture muscle and skin, and put the operated mice back into the cage. Pick up TA muscle at different time points (after denervation 1 day, 3 days, 5 days).

### Compound muscle action potential recording (CMAPs recoding)

Compound muscle action potential recording was performed as described previously [35, 60, 73]. Mice were anesthetized with isoflurane continuously supplied by VetFlo anesthesia system (Kent Scientific) and placed on a 37 °C heating pad. One recording needle electrode (TECA, 092-DMF25-S) was inserted into the middle of the TA or GA muscle, another one as a reference needle electrode was inserted into the Achilles tendon, both of which were connected to AxoPatch 700B Amplifier (Molecular Devices). And two stimulation needle electrodes were inserted and close the sciatic nerve, and connected to an isolator (ISO-Flex, AMPI). The sciatic nerve was stimulated with a series of 10 stimulations (5 V, 0.2 ms) at 1, 5, 10, 20, and 40 Hz, and CMAP was recorded by Digidata1500B and analyzed by Clampfit 10.7 software (Molecular Devices).

#### Electrical stimulation of muscle

Mice were anesthesia with isoflurane continuously supplied by Low-Flow Anesthesia System (Kent Scientific, Torrington, USA) and placed on a 30–35 ℃ plate (Aurora Scientific 1300A) to maintain the body temperature. Stimulation electrodes were inserted into the TA muscle, stimulated with a series pulse (100 mA, 0.2 ms, 1 s, 5 Hz pulse for 50 times with a 10 s delay between each pulse) for 30 days by Aurora Scientific 1300A.

### In vivo tetanic force measurement

Torque muscle tension analysis was performed on mice as described previously [74]. Briefly, mice were anesthetized with isoflurane continuously supplied by VetFlo anesthesia system (Kent Scientific) and placed on a 37 °C heating pad. The knee was fixed on the clamp. The skin on the calf was cut to expose the TA muscle. The distal tendon of TA muscle was isolated with sharp tweezers and tied with a thin thread hanging onto the footplate connected to the servomotor (Aurora Scientific 1300A). The angle between the footplate and the tibia/fibula was usually at 90°. For muscle stimulation, two-needle electrodes were inserted subcutaneously into TA muscle. Firstly, identify the best stimulation strength (C_BS_ mA), a single electrical stimulation (0.2 ms pulse width) was given starting from 100 mA, the muscle force was measured for every 30 mA increase, with an interval of 30 s. When the muscle force was no longer increasing, the current was the best stimulation strength. And then, the best position of muscle contraction was found by adjusting the distance between the footplate and the clamp and measuring the muscle force by stimulating muscle with a single electrical stimulation (C_BS_ mA, 0.2 ms pulse width). When the muscle force was no longer increasing, the position was the best position of muscle contraction. Then, under the best position and the best stimulation strength, the tetanic force was measured by stimulating muscle with a series of stimulations (C_BS_ mA, 0.2 ms pulse width, 300 ms duration) at 25, 50, 75, 100, 125, 150, and 175 Hz, with an interval of 2 min. Tetanic forces were was recorded by DMCv5.500 and analyzed by DMAv5.300, normalized by body weight, and described as N/Kg.

### Statistical analysis

Statistics were computed using GraphPad software. Data with two groups were analyzed using two-tailed paired or un-paired Student’s t-test. For datasets with repeated measures and more than two groups, one-way or two-way ANOVA was used, followed by post hoc Bonferroni or Tukey’s multiple comparison correction. Statistical significance was accepted at the 5% significance level (p < 0.05). Unless otherwise indicated, > 5 mice per group or four or more dishes of muscle cells of one mouse were studied. Data are expressed as means ± SEM.

## Supplementary Information

**Additional file 1: Figure S1.** Characterization of MuSK K608A mice. a, Diagrams of MuSK gene. Lys608 (K608, AAG) mutants to Analine (A, GCA). b, Genotyping by sequencing with revise primer. c, Western blot showing protein level of MuSK and phosphorylated-MuSK using anti-MuSK antibody and anti-phosphotyrosine (4G10) antibody. d, Diaphragms of E17 mice of indicated genotypes were stained mount with α-BTX (red) and SYN/NF (green). Scale bar, 1 mm. **Figure S2.** No effect for synaptic nuclei aggregation in denervated muscle. Single fibers of EDL muscle were isolated with 0.2% collagenase type I and stained α-BTX (red) and DAPI (blue). a, Representative images of synaptic nuclei of denervated EDL muscles. Scale bar, 50 µm. b, Quantification of the number of synaptic nuclei. n = 10 muscle fibers per group, F(4,45) = 0.2245. One-way ANOVA followed by Tukey’s multiple comparisons test. Data were shown as mean ± SEM. **Figure S3.** The role of μ-conotoxin. a, No change of CMAP amplitude ratio 10th/1th in μ-conotoxin treated TA muscle. n = 4 mice per group, F(1,30) = 3.665. p = 0.0651. b, Representative trace of the 1th stimulated CMAP. c, Quantification of the 1th stimulated CMAP amplitude, n = 4 mice per group, ***p = 0.0003. d, Scheme of measuring TA muscle tetanic force. e, Decreased tetanic force under different frequency stimulation in μ-conotoxin treated TA muscle. n = 3 mice per group, F(1,28) = 818.1, ****p < 0.0001. f, Representative trace of tetanic force at 100Hz. a, e Two-way ANOVA followed by Bonferroni’s multiple comparisons test; c, Two-tailed Independent Student’s t-test. Data were shown as mean ± SEM.

## Data Availability

The datasets used and/or analysed during the current study are available from the corresponding author on reasonable request.

## Abbreviations

α-BTX: Alpha-bungarotoxin
β-gal: β-Galactosidase
CMAP: Compound muscle action potential recording
EDL: Extensor digitorum longus
ECD: Extracellular domain
ICD: Intracellular domain
MuSK kd: MuSK kinase-dead
NF: Neurofilament
NMJ: Neuromuscular junction
qPCR: Quantitative polymerase chain reaction
SYN: Synapsin
TA: Tibialis anterior
WT: Wild-type
