# Supplementary figures and images for: Synapse-specific Lrp4 mRNA enrichment requires Lrp4/MuSK signaling, muscle activity and Wnt non-canonical pathway
Source: Cell Biosci. 2021 Jun 5;11:105. doi: 10.1186/s13578-021-00619-z (PMC8180081; doi:10.1186/s13578-021-00619-z)

**Figure S1**

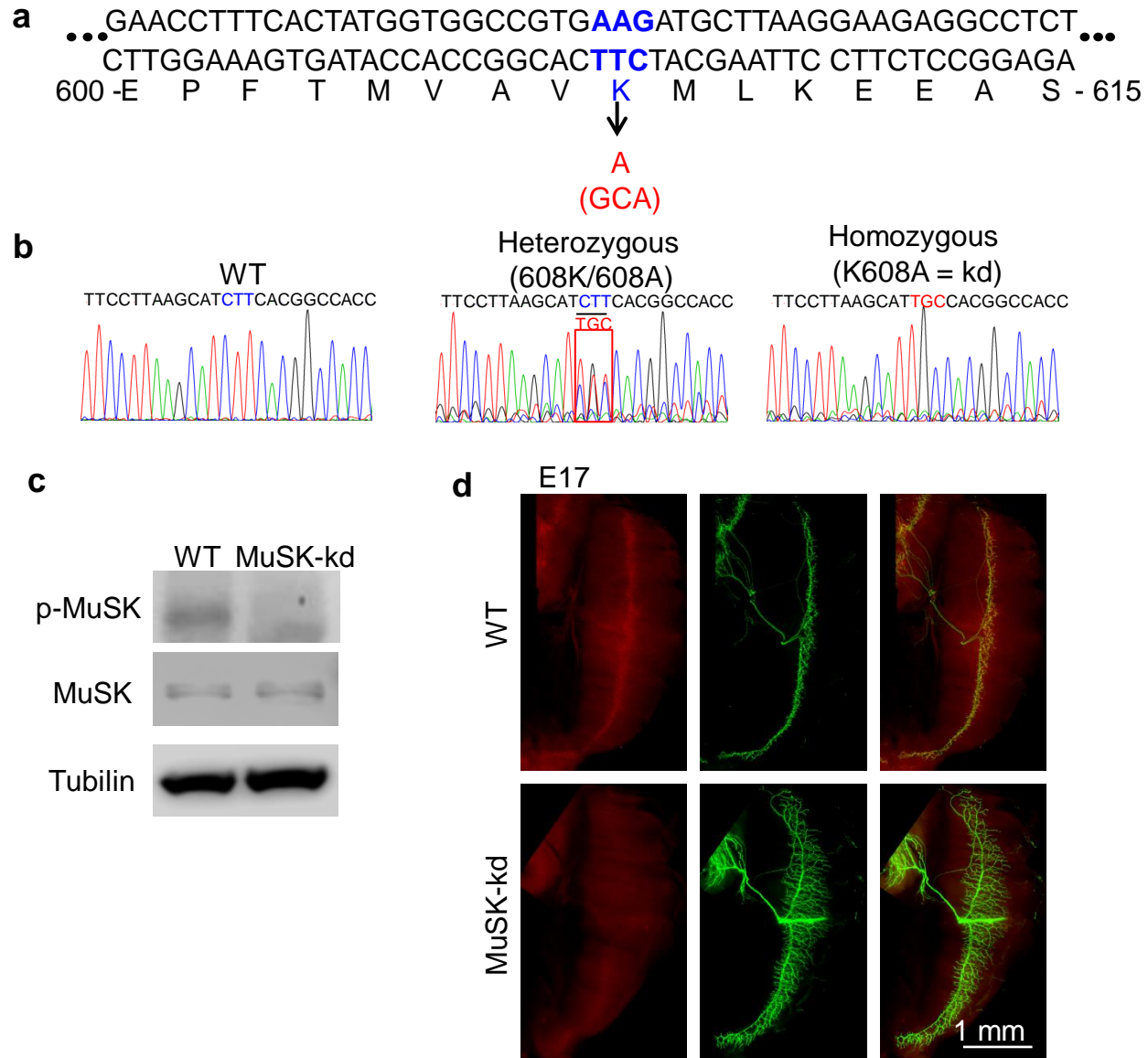

**Figure S2**

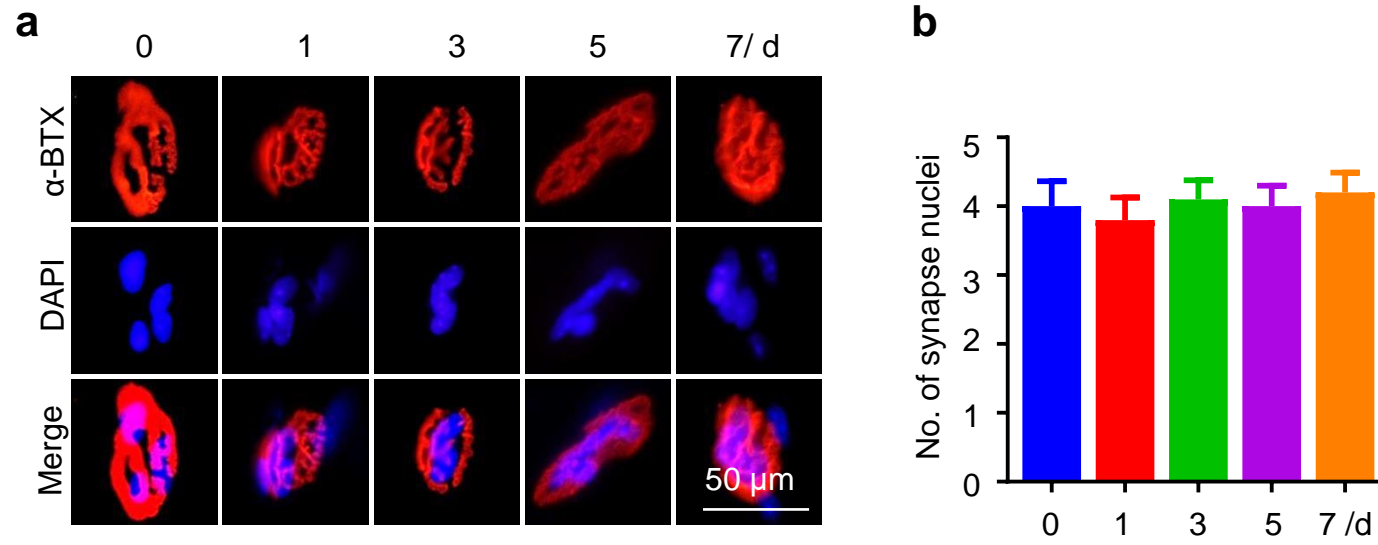

**Figure S3**

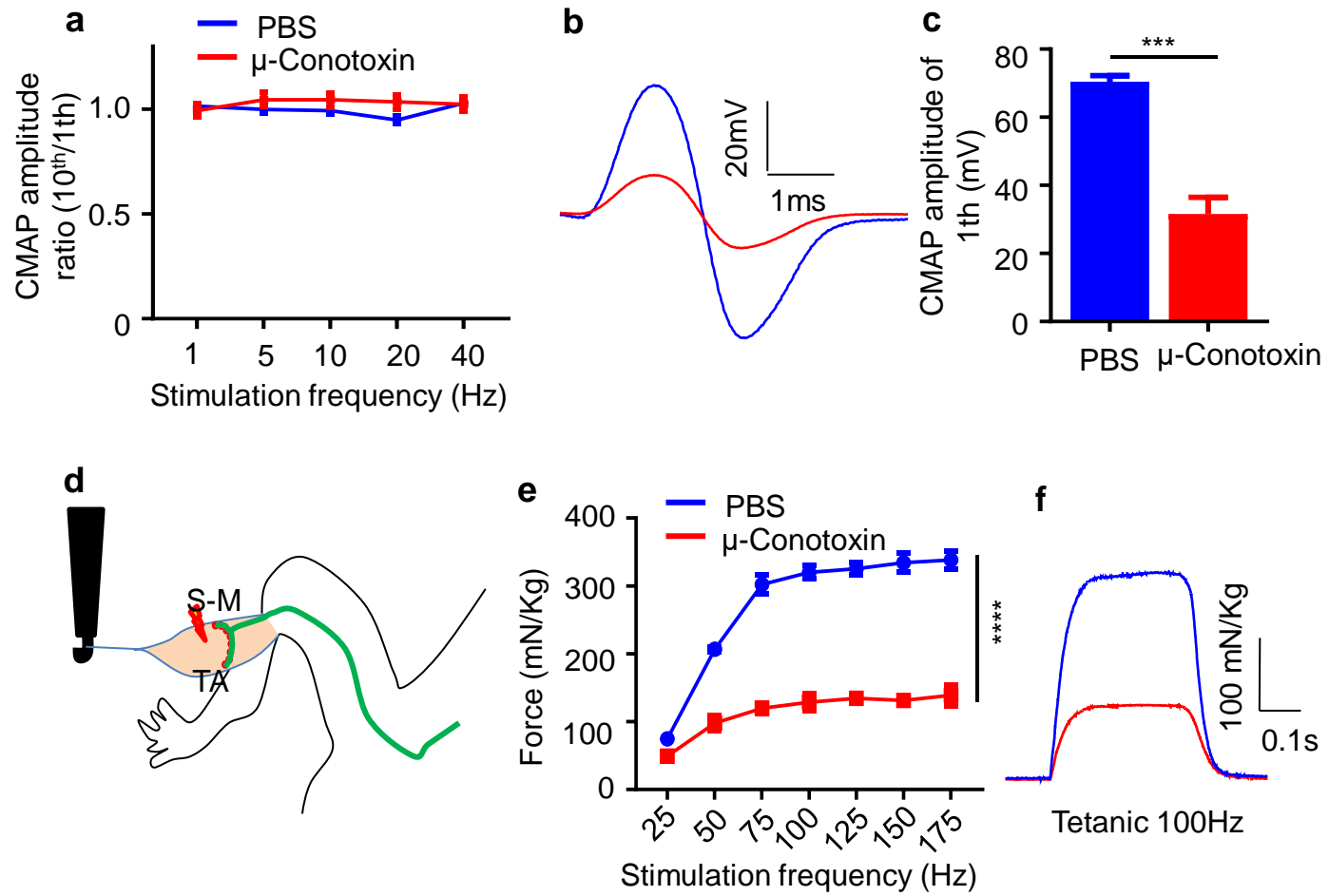

Supplement: Supplementary file 1 — Additional file 1: Figure S1. Characterization of MuSK K608A mice. a, Diagrams of MuSK gene. Lys608 (K608, AAG) mutants to Analine (A, GCA). b, Genotyping by sequencing with revise primer. c, Western blot showing protein level of MuSK and phosphorylated-MuSK using anti-MuSK antibody and anti-phosphotyrosine (4G10) antibody. d, Diaphragms of E17 mice of indicated genotypes were stained mount with α-BTX (red) and SYN/NF (green). Scale bar, 1 mm. Figure S2. No effect for synaptic nuclei aggregation in denervated muscle. Single fibers of EDL muscle were isolated with 0.2% collagenase type I and stained α-BTX (red) and DAPI (blue). a, Representative images of synaptic nuclei of denervated EDL muscles. Scale bar, 50 µm. b, Quantification of the number of synaptic nuclei. n = 10 muscle fibers per group, F(4,45) = 0.2245. One-way ANOVA followed by Tukey’s multiple comparisons test. Data were shown as mean ± SEM. Figure S3. The role of μ-conotoxin. a, No change of CMAP amplitude ratio 10th/1th in μ-conotoxin treated TA muscle. n = 4 mice per group, F(1,30) = 3.665. p = 0.0651. b, Representative trace of the 1th stimulated CMAP. c, Quantification of the 1th stimulated CMAP amplitude, n = 4 mice per group, ***p = 0.0003. d, Scheme of measuring TA muscle tetanic force. e, Decreased tetanic force under different frequency stimulation in μ-conotoxin treated TA muscle. n = 3 mice per group, F(1,28) = 818.1, ****p < 0.0001. f, Representative trace of tetanic force at 100Hz. a, e Two-way ANOVA followed by Bonferroni’s multiple comparisons test; c, Two-tailed Independent Student’s t-test. Data were shown as mean ± SEM. [file 13578_2021_619_MOESM1_ESM.pdf]
